# Supplementary material for: Efficacy and Safety of Different Courses of Tongxinluo Capsule as Adjuvant Therapy for Coronary Heart Disease after Percutaneous Coronary Intervention: A Systematic Review and Meta-Analysis of Randomized Controlled Trials
Source: J Clin Med. 2022 May 25;11(11):2991. doi: 10.3390/jcm11112991 (PMC9181557; doi:10.3390/jcm11112991)
Supplement: Supplementary file 1 [file jcm-11-02991-s001.zip › Supplementary File S1. Search strategies.pdf]

**Search strategies**

| Databases | Terms |
|---|---|
| The Cochrane Library | #1 (*Tongxinluo)<br>#2 (Tong-xin-luo)<br>#3 (Tong Xin Luo)<br>#4 (TXL)<br>#5 (Tongxinluo)<br>#6 #1 OR #2 OR #3 OR #4 OR #5 |
| PubMed | #1 (((Tongxinluo) OR (TXL)) OR (Tong-xin-luo)) OR (Tong Xin Luo)<br>#2 (((Randomized Controlled Trial[ptyp])) OR ((Controlled Clinical Trial[ptyp])) OR ((Clinical Trial[ptyp])) OR (“Clinical Trials as Topic”[Mesh]) OR (“Clinical Trials, Phase III as Topic”[Mesh]) OR (“Clinical Trials, Phase IV as Topic”[Mesh]) OR (“Controlled Clinical Trials as Topic”[Mesh]) OR (“Clinical Trial”[Publication Type]) OR (“Controlled Clinical Trial”[Publication Type]) OR (“Clinical Trial, Phase III”[Publication Type]) OR (“Clinical Trial, Phase IV”[Publication Type]) OR (“Multicenter Study”[Publication Type]) OR (“Multicenter Studies as Topic”[Mesh]) OR (“Random Allocation”[Mesh]) OR (“Double-Blind Method”[Mesh]) OR (“Single-Blind Method”[Mesh]) OR (“Cross-Over Studies”[Mesh]) OR (“Placebos”[Mesh]) OR (controlled[tiab] AND (trial[tiab] OR trials[tiab] OR study[tiab] OR studies[tiab])) OR (blind[tiab] OR blinding[tiab] OR blinded[tiab] OR mask[tiab] OR masking[tiab] OR masked[tiab] OR placebo[tiab] OR placebos[tiab] OR rct[tiab] OR random[tiab] OR randomised[tiab] OR randomized[tiab] OR randomly[tiab] OR randomisation[tiab] OR randomization[tiab]) OR (factorial[tiab]) OR (divided[tiab] AND (group[tiab] OR groups[tiab])) OR (crossover[tiab]) OR (“cross over”[tiab]) OR (multicentre[tiab] OR multicentred[tiab] OR multicentric[tiab]) OR (versus[ti] OR vs[ti]) OR (“treatment arm”[tiab]) OR (“phase III”[tiab] OR “phase three”[tiab] OR “phase 3”[tiab]) OR (“latin square”[tiab]) NOT ((“Animals”[Mesh] OR mouse[ti] OR mice[ti] OR pig[ti] OR pigs[ti] OR rat[ti] OR rats[ti] OR rabbit*[ti]) NOT ((“Animals”[Mesh] OR mouse[ti] OR mice[ti] OR pig[ti] OR pigs[ti] OR rat[ti] OR rats[ti] OR rabbit*[ti] OR cadaver[ti] OR cadavers[ti]) AND (“Humans”[Mesh]))))<br>#3 #1AND#2 |
| Embase | #1 ‘tongxinluo’/exp OR ‘txl’:ab,ti OR ‘tong xin luo’:ab,ti OR ‘tong-xin-luo’:ab,ti |
| China National Knowledge Infrastructure | (SU % = ‘通心络’ OR TI = ‘通心络’ OR KY = ‘通心络’ OR AB = ‘通心络’ OR FT = ‘通心络’ OR SU % = ‘通新络’ OR TI = ‘通新络’ OR KY = ‘通新络’ OR AB = ‘通新络’ OR FT = ‘通新络’ OR SU % = ‘通欣络’ OR TI = ‘通欣络’ OR KY = ‘通欣络’ OR AB = ‘通欣络’ OR FT = ‘通欣络’ OR SU % = ‘痛心络’ OR TI = ‘痛心络’ OR KY = ‘痛心络’ OR AB = ‘痛心络’ OR FT = ‘痛心络’ OR SU % = ‘痛心洛’ OR TI = ‘痛心洛’ OR KY = ‘痛心洛’ OR AB = ‘痛心洛’ OR FT = ‘痛心洛’) AND (SU % = ‘介入治疗’ OR AB = ‘介入治疗’ OR KY = ‘介入治疗’ OR FT = ‘介入治疗’ OR TI = ‘介入治疗’ OR SU % = ‘介入手术’ OR AB = ‘介入手术’ OR KY = ‘介入手术’ OR FT = ‘介入手术’ OR TI = ‘介入手术’ OR SU % = ‘经皮冠状动脉’ OR AB = ‘经皮冠状动脉’ OR KY = ‘经皮冠状动脉’ OR FT = ‘经皮 |

| | |
|---|---|
| | 冠状动脉’ OR TI = ‘经皮冠状动脉’ OR SU % = ‘冠状动脉介入’ OR KY = ‘冠状动脉介入’ OR TI = ‘冠状动脉介入’ OR FT = ‘冠状动脉介入’ OR AB = ‘冠状动脉介入’ OR SU % = ‘冠脉介入’ OR KY = ‘冠脉介入’ OR TI = ‘冠脉介入’ OR FT = ‘冠脉介入’ OR AB = ‘冠脉介入’ OR SU % = ‘冠脉成型’ OR KY = ‘冠脉成型’ OR TI = ‘冠脉成型’ OR FT = ‘冠脉成型’ OR AB = ‘冠脉成型’ OR SU % = ‘冠脉成形’ OR KY = ‘冠脉成形’ OR TI = ‘冠脉成形’ OR FT = ‘冠脉成形’ OR AB = ‘冠脉成形’ OR SU % = ‘冠状动脉成形’ OR KY = ‘冠状动脉成形’ OR TI = ‘冠状动脉成形’ OR FT = ‘冠状动脉成形’ OR AB = ‘冠状动脉成形’ OR SU % = ‘冠状动脉成型’ OR KY = ‘冠状动脉成型’ OR TI = ‘冠状动脉成型’ OR FT = ‘冠状动脉成型’ OR AB = ‘冠状动脉成型’ OR SU % = ‘经皮经管冠状动脉’ OR KY = ‘经皮经管冠状动脉’ OR TI = ‘经皮经管冠状动脉’ OR FT = ‘经皮经管冠状动脉’ OR AB = ‘经皮经管冠状动脉’ OR SU % = ‘经皮经管冠脉’ OR KY = ‘经皮经管冠脉’ OR TI = ‘经皮经管冠脉’ OR FT = ‘经皮经管冠脉’ OR AB = ‘经皮经管冠脉’ OR SU % = ‘经皮腔内冠脉’ OR KY = ‘经皮腔内冠脉’ OR TI = ‘经皮腔内冠脉’ OR TI = ‘经皮腔内冠脉’ OR FT = ‘经皮腔内冠脉’ OR AB = ‘经皮腔内冠脉’ OR SU % = ‘经皮腔内冠状动脉’ OR KY = ‘经皮腔内冠状动脉’ OR TI = ‘经皮腔内冠状动脉’ OR FT = ‘经皮腔内冠状动脉’ OR AB = ‘经皮腔内冠状动脉’ OR SU % = ‘支架’ OR KY = ‘支架’ OR TI = ‘支架’ OR FT = ‘支架’ OR AB = ‘支架’ OR SU % = ‘PCI’ OR KY = ‘PCI’ OR TI = ‘PCI’ OR FT = ‘PCI’ OR AB = ‘PCI’) AND (SU % = ‘临床试验’ OR SU % = ‘随机对照试验’ OR AB = ‘随机’ OR AB = ‘随机对照’ OR AB = ‘临床试验’ OR AB = ‘临床研究’ OR AB = ‘临床观察’ OR AB = ‘临床效果’ OR AB = ‘临床分析’ OR AB = ‘临床疗效’ OR AB = ‘临床比较’ OR AB = ‘对照研究’ OR AB = ‘对照试验’ OR AB = ‘对照治疗’ OR AB = ‘对照观察’ OR AB = ‘对照分析’ OR AB = ‘对比研究’ OR AB = ‘对比观察’ OR AB = ‘对比分析’ OR AB = ‘分组研究’ OR AB = ‘比较研究’ OR AB = ‘多中心研究’ OR AB = ‘疗效观察’ OR AB = ‘疗效评价’ OR AB = ‘疗效分析’ OR AB = ‘疗效比较’ OR AB = ‘治疗研究’ OR AB = ‘治疗比较’ OR AB = ‘效果比较’ OR AB = ‘盲法’ OR AB = ‘双盲’ OR AB = ‘单盲’ OR AB = ‘安慰剂’ OR FT = ‘随机’) |
| Wanfang Database | 题名或关键词:(“介入治疗” or “介入手术” or “经皮冠状动脉” or “冠状动脉介入” or “冠脉介入” or “冠脉成型” or “冠脉成形” or “冠状动脉成形” or “冠状动脉成型” or “经皮经管冠状动脉” or “经皮经管冠脉” or “经皮腔内冠状动脉” or “经皮腔内冠脉” or “支架” or “PCI”) and (“通心络” or “通新络” or “通欣络” or “通心洛” or “痛心洛”) |
| Chinese Scientific Journal Database | M=(介入治疗 OR 介入手术 OR 经皮冠状动脉 OR 冠状动脉介入 OR 冠脉介入 OR 冠脉成型 OR 冠脉成形 OR 冠状动脉成形 OR 冠状动脉成型 OR 经皮经管冠状动脉 OR 经皮经管冠脉 OR 经皮腔内冠状动脉 OR 经皮腔内冠脉 OR 支架 OR PCI) AND M=(通心络 OR 通新络 OR 通欣络 OR 通心洛 OR 痛心洛) |

# indicates the step number.
